# Chromosomal and plasmid-mediated fluoroquinolone resistance in human *Salmonella enterica* infection in Ghana

**DOI:** 10.1186/s12879-019-4522-1

**Published:** 2019-10-28

**Authors:** Godfred Acheampong, Michael Owusu, Alex Owusu-Ofori, Isaac Osei, Nimako Sarpong, Augustina Sylverken, Hung-Jui Kung, Shu-Ting Cho, Chih-Horng Kuo, Se Eun Park, Florian Marks, Yaw Adu-Sarkodie, Ellis Owusu-Dabo

**Affiliations:** 1 Kumasi Centre for Collaborative Research in Tropical Medicine, Kumasi, Ghana; 2 Department of Medical Laboratory Technology, Kwame Nkrumah University of Science and Technology, Kumasi, Ghana; 3 Department of Clinical Microbiology, Kwame Nkrumah University of Science and Technology, Kumasi, Ghana; 4 Komfo Anokye Teaching Hospital, Kumasi, Ghana; 5 Agogo Presbyterian Hospital, Agogo, Ghana; 6 Department of Theoretical and Applied Biology, Kwame Nkrumah University of Science and Technology, Kumasi, Ghana; 7 Institute of Plant and Microbial Biology, Academia Sinica, Taipei, Taiwan; 8 Department of Epidemiology, International Vaccine Institute, Seoul, South Korea; 9 Oxford University Clinical Research Unit, Wellcome Trust Major Overseas Programme, 764 Vo Van Kiet, Quant 5, Ho Chi Minh City, Vietnam; 10 Department of Global and International Health, School of Public Health, Kwame Nkrumah University of Science and Technology, Kumasi, Ghana

**Keywords:** Fluoroquinolone resistance, Plasmids, *Salmonella enterica*, Mutations

## Abstract

**Background:**

*Salmonella* infection poses significant public health threat globally, especially in resource-limited countries. Emergence and spread of antibiotic resistant strains to fluoroquinolones have led to treatment failures and increased mortality in *Salmonella* infection. However, there is dearth of information regarding mechanisms of resistance to fluoroquinolones in Ghana. This study therefore sought to identify chromosomal mutations and plasmid-mediated resistance as possible mechanisms of fluoroquinolone resistance from clinical isolates in Ghana.

**Methods:**

This was a retrospective study of archived isolates biobanked at Kumasi Centre for Collaborative Research in Tropical Medicine, Ghana. Isolates were obtained from blood, stool and oropharynx samples at two hospitals, between May, 2016 and January, 2018. *Salmonella* identification was done using standard microbiological protocols and antibiotic susceptibility testing performed by Kirby-Bauer disc diffusion method. Isolates with intermediate susceptibility and/or resistance to nalidixic acid and/or ciprofloxacin were selected and examined for chromosomal mutations by Sanger sequencing and plasmid-mediated resistance by PCR.

**Results:**

Of 133 biobanked isolates cultured, 68 (51.1%) and 16 (12%) were identified as *Salmonella* Typhi and non-typhoidal *Salmonella* (NTS), respectively. Sequence analysis of *gyrA* gene revealed the presence of 5 different nonsynonymous mutations, with the most frequent mutation (Ile203Ser) occurring in 12 out of 13 isolates tested. Gyrase B (*gyrB*) gene had 1 nonsynonymous mutation in 3 out of 13 isolates, substituting phenylalanine with leucine at codon 601 (Phe601Leu). No mutation was observed in *parC* and *parE* genes. Two NTS isolates were found to harbour *qnrS* plasmid-mediated resistant gene of molecular size 550 bp with high ciprofloxacin MIC of 0.5 μg/ml.

**Conclusion:**

This study reports for the first time in Ghana plasmid-mediated fluoroquinolone resistant gene *qnrS* in *Salmonella* clinical isolates. Nonsynonymous mutations of *gyrA* and *gyrB* genes likely to confer *Salmonella* reduced susceptibility to ciprofloxacin were also reported.

## Background

Human *Salmonella enterica* infection poses a significant public-health challenge globally, especially in low-to-middle income countries in sub-Saharan Africa and South Asia where sanitation is poor [1]. The situation is worsened by an increasing rate of emergence and distribution of antibiotic resistant strains [2–4]*.*

One surveillance study has demonstrated an obvious increase in overall antimicrobial resistance from 20 to 30% in the early 1990s to as high as 70% in the early 2000s [5]. This report was based on old generic antibiotics which are not currently in use. Chloramphenicol, ampicillin and sulfamethoxazole trimethoprim used to be the drugs of choice for treating *Salmonella* infection for over a decade [6] . However, because of increased resistance to these first-line drugs, physicians have resorted to ciprofloxacin (a fluoroquinolone) [7]. Since its introduction, there has not been structured surveillance mechanism(s) to identify mutations possibly associated with its resistance in Ghana. Availability of only few new antibiotics, has placed enormous value on investigations into resistance strains of bacteria. The World Health Organisation (WHO) has listed fluoroquinolone–resistant *Salmonella* spp. as part of the priority pathogens for which new antibiotics are urgently needed [8]. Although phenotypic resistance could be available in some clinical laboratories, there is paucity of data on molecular investigations of fluoroquinolone resistant genes associated with *Salmonella* infection in Ghana and many African countries.

Fluoroquinolone resistance is mainly due to two mechanisms: chromosomally mediated mutations occurring at the quinolone resistance determining regions (QRDR) of topoisomerase genes (*gyrA, gyrB, parC* and *parE* genes) and resistance mediated by plasmids [9]. The latter is further divided into 3 different routes that confer decreased susceptibility to fluoroquinolones: 1) quinolone resistance proteins (encoded by *qnr* genes (*qnrA, qnrB, qnrC, qnrD, qnrS*) that shield DNA gyrase from the effect of fluoroquinolones); 2) aac(6′)-Ib-cr resistance mechanism (an aminoglycoside acetyltransferase that modifies fluoroquinolones by acetylating the free nitrogen on the C7 ring of the drug, decreasing binding affinity), and 3) plasmid-mediated resistance by OqxAb and QepA efflux systems [10, 11].

Fluoroquinolones resistance mediated by plasmids have been identified in some developed countries such as United States of America, United Kingdom and France [9, 12, 13]. With the high rate of spread of plasmid-mediated resistance determinants coupled with some factors such as international travels, there is a possibility of fluoroquinolone resistant *Salmonella* strains circulating in Ghana. More often than not, fluoroquinolone resistance in *Salmonella enterica* is mediated by *gyrA* mutations [3, 14], with few reported cases of *gyrB* mutations [15] and very few cases of topoisomerase IV (*parC* and *parE*) genes. Mutation results in a significant reduction of the drug-enzyme binding, and as such the ability for fluoroquinolones to inhibit DNA ligation is totally restricted [16, 17]. Both chromosomal and plasmid-encoded fluoroquinolone resistance are responsible for conferring low-level resistance to fluoroquinolones [18], nonetheless, high level-resistance (with increasing MIC up to 250-fold) has been documented [19].

Fluoroquinolone resistant *Salmonella* strains with multiple gyr and par mutations have been reported from Cambodia, India and Nepal [20, 21]. In Africa, fluoroquinolone resistant strains of *Salmonella* are known to circulate in countries such as Kenya, Tanzania, Malawi, South Africa, Zambia, Democratic Republic of Congo and Nigeria [4, 22, 23]. However, meta analysis conducted by Cuypers et al.*,* revealed lower prevalence and spread of these strains in Africa compared to Asia [8].

This study therefore sought to identify resistance associated with mutations in the topoisomerase genes of *Salmonella* and plasmid-mediated resistant genes associated with fluoroquinolone resistance in *Salmonella* strains from Ghana.

## Methods

### Study design and study area

This was a retrospective study of archived isolates (from blood, stool and oropharynx samples) biobanked at Kumasi Centre for Collaborative Research in Tropical Medicine (KCCR), Ghana, between May, 2016 and January, 2018. These isolates were collected as part of a larger study that sought to investigate the burden of severe typhoid in sub-Saharan Africa with six countries in participation (Ghana, Burkina Faso, Democratic Republic of Congo, Ethiopia, Nigeria and Madagascar). Nonetheless, this report focused only on the Ghanaian site. Study population comprised patients that presented with fever at Komfo Anokye Teaching Hospital (KATH) and Agogo Presbyterian Hospital (APH) in the Kumasi Metropolis and Asante-Akim North district, respectively, both located in the middle belt of Ghana. KATH serve as a tertiary hospital in an urban setting whereas APH is a primary health facility in a rural area of Ghana.

### Ethics approval

The main study protocol was reviewed and approved by the Committee for Human Research Publications and Ethics (CHRPE) at the School of Medical Sciences, Kwame Nkrumah University of Science and Technology (KNUST) (Approval Number: CHRPE/AP/188/18).

### Data collection

Study participants’ biodata such as age and gender were collected from the data department of KCCR. Information regarding the source of isolates, total number of samples received and processed were retrieved from laboratory data.

### Laboratory procedures

#### Bacterial culture

Biobanked isolates were removed from − 80 °C freezer (Thermo Scientific), thawed and sub-cultured unto three standard growth media: Blood Agar (BA – Columbia agar base supplemented with 5% sheep blood), chocolate agar (CA) and macConkey agar (Mac) (BD, Franklin Lakes, New Jersey, USA) under sterile working condition. All the plates were incubated aerobically overnight at 35 °C–37 °C except for CA plates which was incubated in 5% CO_2_ for microaerophilic condition.

#### Bacterial identification

*Salmonella* was identified based on colonial morphology on the various agar, microscopic presentation, latex agglutination test, biochemical tests (including API20E), as well as serotyping (by White-Kauffmann Le-Minor scheme) using commercially available serotyping kit from BD (Franklin Lakes, New Jersey, USA). On both the BA and CA, small, creamy gamma (ɣ) hemolytic colonies consistent with *Salmonella* sp. was recorded. Gram negative short rods with small colourless non-lactose fermenting colonies on Mac was also documented. Biochemical tests such as triple sugar iron (TSI), urease, citrate tests were performed to aid in *Salmonella* identification from other enterobacteria based on sugars fermentation, urease production and citrate utilisation, respectively. Isolation and identification of other gram negative and positive bacteria were done according to standard microbiological protocols.

#### Antibiotic susceptibility testing

We performed antimicrobial susceptibility testing on all biobanked isolates confirmed as *Salmonella* according to Clinical and Laboratory Standards Institute (CLSI) guidelines [24]. Susceptibility to ampicillin (10 μg), amoxiclav (amoxicillin & clavulanic acid; 20/10 μg), ceftriaxone (30 μg), trimethoprim/sulfamethoxazole (1.25/23.75 μg), ciprofloxacin (5 μg), gentamicin (10 μg), tetracycline (30 μg), chloramphenicol (30 μg), ceftazidime (30 μg), cefotaxime (30 μg) and nalidixic acid (30 μg) was tested on Mueller Hinton agar (BD, USA) using the Kirby-Bauer disc diffusion method. The breakpoints of the various antibiotics used were in line with CLSI 2018. Resistance to fluoroquinolones, defined as isolates with intermediate susceptibility and/or resistance to nalidixic acid (surrogate marker for ciprofloxacin resistance) and/or ciprofloxacin were selected for Minimum Inhibitory Concentration (MIC). Enterobacteria such as *E. coli* and *Klebsiella* sp. that were resistant to 3rd generation cephalosporins in this study were further screened to detect the presence of extended spectrum beta-lactamase (ESBL) enzyme using double-disc diffusion method on Mueller Hinton agar according to CLSI guidelines [24]. Again, *S. aureus* resistant to cefoxitin were regarded as methicillin-resistant *Staphylococcus aureus* (MRSA).

#### MIC determination

Minimum inhibitory concentration (MIC) was performed on ciprofloxacin/nalidixic acid intermediate and/or resistant isolates using ciprofloxacin E-test (epsilometer test) according to the manufacturers recommendation (Oxoid, Wesel, Germany) to confirm ciprofloxacin resistance. E-test gives a direct quantification of antimicrobial susceptibility in the form of discrete MIC values. Isolates with ciprofloxacin breakpoint concentration (μg/ml) of ≤0.06 μg/ml was documented as sensitive (S); between 0.12 and 0.5 μg/ml as intermediate (I); and ≥ 1 μg/ml was reported as resistant (R) following the CLSI guidelines.

#### Quality control

*Escherichia coli* ATCC 25922 and *Salmonella* Typhimurium ATCC 14028 were set up together with the test organisms to control media, biochemical tests, potency of antibiotic discs, and ciprofloxacin E-test strip.

### Molecular detection of fluoroquinolone resistant genes

#### DNA extraction

Genomic DNA was extracted from ciprofloxacin and/or nalidixic acid resistant and/or intermediate isolates using spherolyse DNA isolation kit (HainLife Science, Nehren, Germany) according to manufacturer’s instructions. Extracted DNA were used as templates for detection of chromosomally-encoded mutations in the topoisomerase genes and plasmid-mediated fluoroquinolone resistant genes.

#### Amplification of topoisomerase genes

Detection and amplification of *gyrA, gyrB, parC* and *parE* genes by PCR was performed using primers shown in Table 1. Twenty-five microlitres of One Taq Quick-Load 2x Master Mix with standard buffer (New England Biolabs® Inc) was added to 1 μl each of 10 μM forward and reverse primers respectively. Twenty-two microlitres of nuclease-free water was added to the mastermix and finally, 1 μl DNA template to obtain a reaction volume of 50 μl.

**Table 1:** Sequence of primers for detection of *gyrA, gyrB, parC* and *parE* genes

Target gene	Nucleotide sequence (5′ -3′)	Product size (bp)	References
*GyrA*	F 5′ –ATGAGCGACCTTGCGAGAGAGAAATACACCG − 3′	632	[25]
	R 5′ – TTCCATCAGCCCTTCAATGCTGAGTCTTC − 3′		
*GyrB*	F 5′ – AAGCGCGATGGCAAAGAAG − 3′	1500	[25]
	R 5′ – AACGGTCTGCTCATCAGAAAGG − 3′		
*ParC*	F 5′- CTATGCGATGTCAGAGCTGG − 3′	270	[26]
	R 5′- TAACAGCAGCTCGGCGTATT − 3′		
*ParE*	F 5′- TCTCTTCCGATGAAGTGCTG − 3′	240	[26]
	R 5′- ATACGGTATAGCGGCGGTAG − 3′		

Amplification using Veriti thermal cycler was conducted using the following PCR cycling condition: an initial denaturation at 94.0 °C for 30 s; 30 cycles of 94.0 °C for 30 s, *60/54/53/52 °C for 1 min and 68.0 °C for 1 min with a final extension of 68.0 °C for 5 min. The reaction was put on hold at 4 °C until attended to.

Note: *60/54/53/52 °C *corresponds to annealing temperatures of gyrA, gyrB, parC and parE genes, respectively.*

#### PCR product purification

The products of amplification of *gyrA, gyrB, parC* and *parE* genes were purified using DNA clean and concentrator™-25 kit (Zymo research, Irvine, USA) according to manufacturer’s instruction. This was to ensure that ultra-pure PCR products are recovered ahead of Sanger sequencing.

#### DNA sequencing and analysis of mutation

Sanger sequencing of the purified PCR products was achieved using the aforementioned primers of the topoisomerase genes on an ABI 3730XL DNA Analyzer. Analysis of the DNA sequences was performed by comparing with the reference *S*. Typhimurium strain LT2 genome (accession number AE006468.2) for *gyrA*, *gyrB*, *parC* and *parE genes* (accession numbers AAL21173.1, AAL22694.1, AAL22048.1 and AAL22055.1, respectively) at GenBank database using the NCBI (National Centre for Biotechnology Information) BLAST (basic local alignment search tool) program. ExPASy (**Ex**pert **P**rotein **A**nalysis **Sy**stem) translate tool, SIB (Swiss Institute of Bioinformatics) was used to translate the nucleotide sequences into amino acid sequences. Global alignment tool EBI (European Bioinformatics Institute) was used to investigate for any mutations using Needleman-Wunsch algorithm (EMBOSS).

#### Detection of plasmid-mediated quinolonone resistance genes (PMQR)

Polymerase chain reaction (PCR) amplification of fluoroquinolones resistant genes: *qnrA, qnrB* and *qnrS* was performed on all *Salmonella* isolates using the primers [9] in Table 2. Genomic DNA extraction and PCR master-mix preparation were prepared as mentioned before.

**Table 2:** Primers used for amplification of PMQR genes

Name	Sequence	Size (bp)	References
QnrA-FW	5′-GGGTAT GGATATTATTGATAAAG-3′	660	[9]
QnrA-RV	5′-CTAATCCG GCAGCACTATTA-3′		
QnrB-FW	5′-GGMATHGAAATTCGCCACTG-3′	264	[9]
QnrB-RV	5′-TTTGCYGYYCGCCAGTCGAA-3′		
QnrS-FW	5′-AGTGATCTCACCTTCACCGC-3′	550	[9]
QnrS-RV	5′-CAGGCTGCAATTTTGATACC-3′		

PCR experiments were carried out according to the following cycling conditions for all three genes: initial denaturation - 94 °C for 30s; template denaturation - 94 °C for 30s; annealing - 55 °C for 60s; extension - 68 °C for 60s; final extension - 68 °C for 5mins; and reaction was put on hold at 4 °C until amplicons were collected for agarose gel electrophoresis.

#### Gel documentation

The amplicons were resolved by agarose gel electrophoresis (1.5% agarose) at 120 V for an hour and band visualisation done with the aid of UV-transilluminator (Vilber Lourmat, Collegien, France). The concentation of agarose used was more suitable for the expected band sizes in this work. The stained gel was captured unto a desktop computer using the infinity® software.

### Statistical analysis

Data were entered into Microsoft excel and exported to STATA version 12 (Stata Corp, USA) for analysis. Descriptive statistics was used to summarize the distribution of various variables into tables and graphs. Differences between discrete variables were analysed using Fisher’s exact test.

## Results

### Socio-demographic characteristics of the study population

Majority (402/1036; 38.8% and 251/364; 69.0%) of the sampled population from the two study sites (APH and KATH) were ≤ 5 years old. The overall mean (±SE) age (in years) of participants was 15.4 ± 0.5. The mean (±SE) age (in years) of recruited patients seeking medical attention at APH and KATH was 18.7 ± 0.6 and 4.5 ± 0.2 respectively. At both sites, there were high proportion of males compared to females.

### Distribution of bacteremic pathogens

**Fig. 1:**
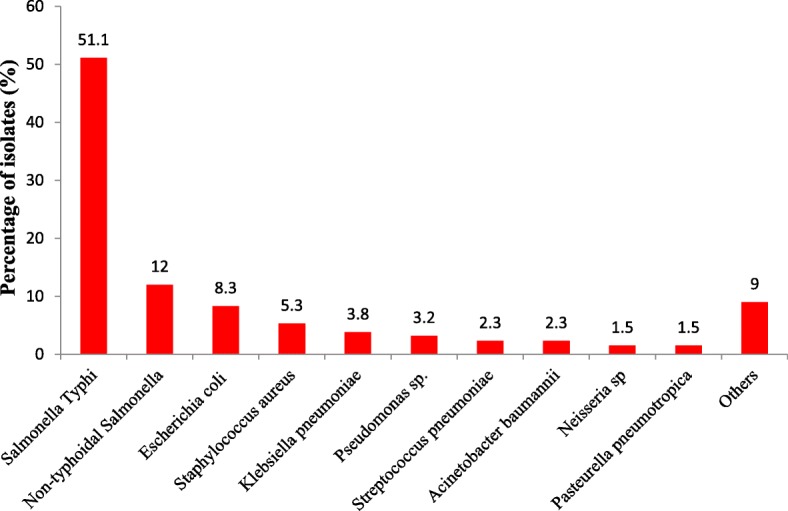
Distribution of bacteremic isolates from study sites. Biobanked bacterial isolates were cultured and identified using standard microbiological culture methods and biochemical tests

Of 133 biobanked isolates cultured, 68 (51.1%) and 16 (12%) were identified as *Salmonella* Typhi and non-typhoidal *Salmonella* (NTS) respectively (Fig. 1). Other bacteriae identified included *Escherichia coli* (including ESBL; 11; 8.3%), *Staphylococcus aureus* (including MRSA; 7; 5.3%) and *Klebsiella pneumoniae* (5; 3.8%)*. Salmonella* Typhi was predominantly found in APH (56/68; 82.4%) while NTS was high in KATH (9/16; 56.3%).

### Salmonella from stool and oropharynx

A total of 13 *Salmonella* strains were isolated from 418 stool specimens collected from both APH and KATH. Of the 13 strains, 4 (30.8%) and 9 (69.2%) were *S.* Typhi and NTS, respectively (Table 3). *Salmonella* was the only pathogen isolated from stool. Five hundred and fifty eight oropharyngeal specimens (OPS) were collected from the two study sites: APH – 401 (71.9%) and KATH – 157 (28.1%). Of the four (4) *Salmonella* strains isolated from 558 OPS, only 1 (25.0%) was identified as *S*. Typhi and 3 (75.0%) NTS (Table 3). All the *Salmonella* strains isolated from stool and OPS were from APH but not KATH.

**Table 3:** Bacterial isolates from stool and oropharynx

Pathogen	n(%) pathogens from	
	Stool	OPS
*S*. Typhi	4 (30.8)	1 (25.0)
NTS	9 (69.2)	3 (75.0)
Total	13 (100)	4 (100)

### Serotyping of bacteremic isolates

Serotyping of iNTS revealed that *Salmonella* Typhimurium (10/16; 62.5%) was the most predominant serovar identified followed by *Salmonella* Enteritidis (5/16; 31.3%) and finally, 1 (1/16; 6.2%) untypable isolate. Rate of isolation of *Salmonella* Typhimurium was significantly higher in APH than in KATH (*p* = 0.011; Table 4), however, *Salmonella* Enteritidis was isolated from only KATH.

**Table 4:** Invasive NTS distribution among study population

Serovar	KATH [n(%)] (*n* = 9)	APH [n(%)] (*n* = 7)	*p*-value
*Salmonella* Typhimurium	3 (18.8)	7 (43.8)	0.011
*Salmonella* Enteritidis	5 (31.1)	0	0.034
Untypable	1 (6.3)	0	–

### Antibiotic resistance profile

Generally, first-line anti-*Salmonella* drugs (ampicillin, chloramphenicol, trimethoprim/sulfamethoxazole) recorded the highest rate of resistance in both *S.* Typhi and NTS isolates (between 33.8 and 50.0%). Third-generation cephalosporins (ceftazidime and ceftriaxone) and gentamicin had 100% efficacy rate in all serovars of *Salmonella* tested. Ciprofloxacin recorded significantly reduced susceptibility (intermediate) in both typhoidal and NTS (14.7 and 37.5%, respectively).

### MIC determination for resistant and reduced-susceptible *Salmonella* strains to fluoroquinolones

Of the 20 ciprofloxacin intermediate/resistant *Salmonella* isolates tested by MIC, only 5 (breakpoint concentration between 0.12 and 0.5 μg/ml) were intermediate and no resistance recorded (Table 5).

**Table 5:** MIC of fluoroquinolone resistant and reduced-susceptible *Salmonella* strains

Isolate number	MIC (μg/ml)	Interpretation
16, 18	0.500	I
1, 14, 15	0.120	I
2, 5, 6, 8, 13, 17, 19, 20	0.030	S
3, 4, 7, 9, 10, 11, 12	0.015	S

*I* intermediate, *S* sensitive

### Identification of mutations within QRDR

Thirteen isolates were selected (based on MIC values from 0.03 to 0.5 μg/ml) for DNA product purification and subsequent sequencing prior to mutational analysis. Sequence analysis of *gyrA* gene revealed the presence of 5 different nonsynonymous mutations, with the most frequent mutation (Ile203Ser) occurring in 12 out of 13 isolates tested (Table 6). Gyrase B (*gyrB*) gene revealed 1 nonsynonymous mutation in 3 out of 13 isolates, substituting amino acid phenylalanine with leucine at codon 601 (Phe601Leu). No mutation was observed in *parC* and *parE* genes. In *gyrA* mutation alone, Serovar Typhi recorded the highest mutation (5/13), followed by Typhimurium (4/13) and Enteritidis (4/13). All three isolates which harboured *gyrB* mutation were *Salmonella* Typhi. Again, 3 isolates, all *S*. Typhi possessed both *gyrA* and *gyrB* mutations. Only one *S*. Enteritidis isolate exhibited Lys154Asn *gyrA* mutation and 5 *S*. Typhi isolates had a Glu133Gly mutation in *gyrA* gene (Table 6). The two isolates with the highest ciprofloxacin MIC (0.5 μg/ml) had common amino acid substitutions resulting in 1 nonsynonymous mutation in *gyrA* gene (Ile203Ser).

**Table 6:** Summary of resistance profiles, target gene mutations and prevalence of PMQR genes

Serovar	Study site	Cip MIC (μg/ml)	Coresistance	Target mutations		
				*gyrA*	*gyrB*	*PMQR*
Typhi	APH	0.12	AMPCSXTNATET	*Ser83Tyr*	*Phe601Leu*	
				*Glu133Gly*		
				*Ile203Ser*		
Typhi	APH	0.03	AMPCSXTTET	*Glu133Gly*	*Phe601Leu*	
				*Ile203Ser*		
Typhi	APH	0.03		*Glu133Gly*		
				*Ile203Ser*		
Typhi	APH	0.03		*Glu133Gly*	*Phe601Leu*	
				*Ile203Ser*		
Typhi	APH	0.03		*Glu133Gly*		
				*Ile203Ser*		
Enteritidis	KATH	0.03	AMPCSXTTET	*Ile203Ser*		
Enteritidis	KATH	0.12		*Asp87Gly*		
				*Ile203Ser*		
Enteritidis	KATH	0.12		*Asp87Gly*		
				*Ile203Ser*		
Enteritidis	KATH	0.50		*Ile203Ser*		*qnrS*
Typhimurium	APH	0.03		*Ile203Ser*		
Typhimurium	APH	0.50	AMPAMC	*Ile203Ser*		*qnrS*
Typhimurium	APH	0.03	AMPAMC	*Lys154Asn*		
Typhimurium	APH	0.03	AMPCSXTAMC	*Ile203Ser*		

### Detection of plasmid-mediated qnr genes

**Fig. 2:**
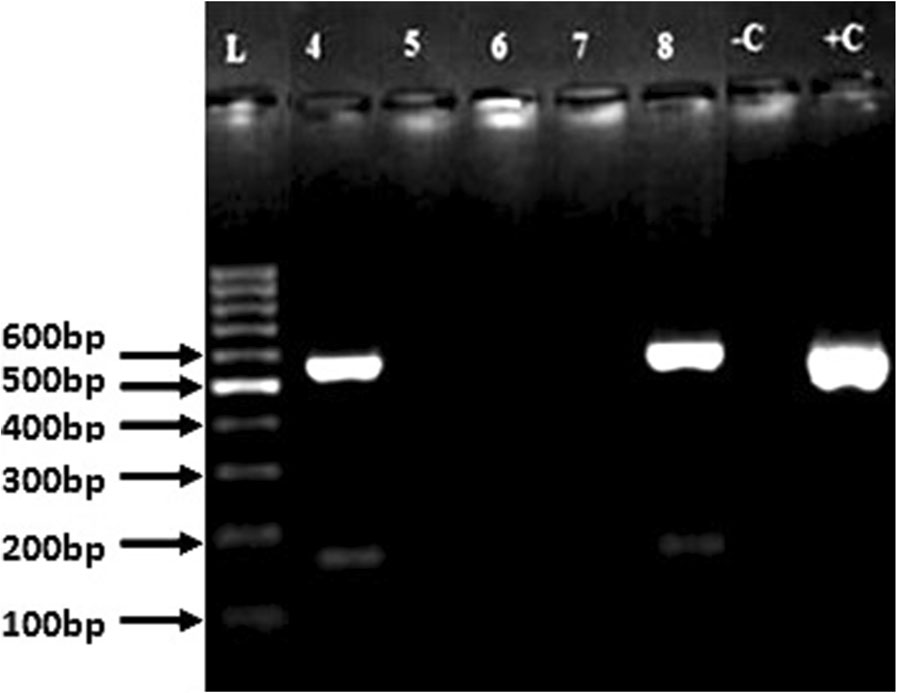
Amplification and detection of *qnrS* plasmid-mediated fluoroquinolone resistant gene (molecular size 550 bp) by PCR. Lanes 4,8 are positive for *qnrS* gene (550 bp). Lanes –C, +C are negative and positive controls respectively.L = Molecular ladder of size 100 bp

Again, Of the 20 phenotypically resistant and/or intermediate *Salmonella* isolates to ciprofloxacin and nalidixic acid tested by singleplex PCR reactions, there was no amplification of *qnrA* and *qnrB* plasmid-mediated resistant genes. However, 2 isolates were found to harbour *qnrS* resistant gene of molecular size 550 bp (Fig. 2). Both isolates were non-typhoidal *Salmonella* strains from the blood (*S.* Enteritidis) and oropharynx (*S.* Typhimurium) of 11 and 1 year old children at KATH and APH, respectively. Again, these two isolates recorded the highest MIC value of 0.5 μg/ml (Table 6).

## Discussion

This study identified five different *gyrA* mutations and one *gyrB* nonsynonymous mutation in quinolone-resistant *Salmonella enterica* from human clinical isolates. *Salmonella* isolates harbouring plasmid-mediated fluoroquinolone resistant gene *qnrS* was also identified in this study.

There was high frequency of *gyrA* gene mutation in this study than the other topoisomerase genes examined. This agrees with findings from Eaves et al.*,* in that mutations occurring at the quinolone resistance determining regions of other topoisomerase genes are more uncommon than those observed in *gyrA* gene [27]. Thus, there might be other mechanisms of resistance playing an important role, as mutations in all but *gyrA* gene were rare. In Africa, the most common mutation known to account for ciprofloxacin non-susceptibility in most clinical *Salmonella* isolates is found in *gyrA* gene, followed by *parC* and *gyrB* genes, with no reported case of *parE* gene mutation [28]. As observed in this study, both *parC* and *parE* gene mutations were completely absent in the study populations, consistent with previous studies [29]. Report by Bae et al., showed a higher frequency (18 out of 27 isolates) of *gyrA* mutation (Asp87Gly) in nalidixic acid resistant *S*. Enteritidis from South Korea [30]. However, the present study identified only 2 nalidixic acid resistant *S*. Enteritidis with the same Asp87Gly *gyrA* mutation and had ciprofloxacin MIC 0.12 μg/ml. Codons 83 and 87 of *gyrA* gene are widely known to be a common hotspot for *gyrA* mutation [31, 32], nevertheless, their frequency was much lower in the current study. Mutations at these codons, especially codon 87, have been shown to be associated with decreased ciprofloxacin susceptibility and nalidixic acid resistance [31].

Other *gyrA* mutation was detected in 5 *S*. Typhi isolates which resulted in amino acid substitution from glutamic acid to glycine at codon 133 with MIC of 0.03 μg/ml (4 isolates) and 0.12 μg/ml (only one isolate). With these low MICs, it could be suggested that mutation of Glu133Gly alone could not necessarily lead to quinolone resistance in *Salmonella*. This agrees with findings from Eibach et al.*,* who detected a Glu133Gly mutation in ciprofloxacin susceptible *S.* Typhi clinical isolate in Ghana with MIC of 0.06 μg/ml [29]. However, studies in Kenya found 11 *S*. Typhi reduced ciprofloxacin susceptible isolates with the same *gyrA* mutation (Glu133Gly) [33].

Studies show that double mutations found in gyrase further reduce binding affinity of the enzyme-DNA complex to fluoroquinolones [34]. This agrees with the current study as 2 *S.* Typhi isolates identified to have double mutations in *gyrA* and *gyrB* genes were MDR and also resistant to the quinolone nalidixic acid.

Gyrase B gene mutation was detected in only 3 *S*. Typhi isolates which resulted in amino acid substitution from phenylalanine to leucine at codon 601 (Phe601leu). The first *gyrB* gene mutation (Glu466Asp) reported in Ghana was from *S*. Typhimurium [15], however, the present study identified *gyrB* mutation in *S*. Typhi, for the first time in Ghana. Findings from Tadesse et al.*,* revealed a low proportion (0.2%) of *Salmonella gyrB* mutation from human and animal sources in Africa [28].

Molecular analysis by PCR detected the presence of quinolone resistance gene *qnrS* in 2 non-typhoidal *Salmonella* isolates from the blood and oropharynx in patients attending health care at KATH and APH respectively. The 3 principal genes responsible for plasmid-mediated fluoroquinolone resistance in *Salmonella* include *qnrA, qnrB* and *qnrS* genes [9]. Previous studies in Ghana showed the absence of all three genes [15, 29], indicating a more recent emergence of *qnrS* resistant gene. Plasmids increase bacterial genetic diversity greatly through acquisition or loss of genes especially, those pertaining to resistance and/or virulence. Detection of *qnrS* gene in this study might be due to indiscriminate use of over-the-counter antibiotics by humans, without complying to clinicians prescriptions. This could lead to increased selective pressure on the drugs and subsequently contributes to resistance. Again, there could be possibility of zoonotic transmission of these resistant genes [35, 36], as Dekker et al.*,* has already reported on emergence of *qnrB* resistant gene in poultry population in Ghana [37].

Variant *qnrA1* of the *qnrA* gene family was the first plasmid-mediated fluoroquinolone resistant gene described, however, several studies show that this gene is not commonly found in *Salmonella* [9, 38, 39]. This might explain why none of the isolates tested in this study was positive for *qnrA*.

Dekker et al.*,* detected *Salmonella* Poona plasmid-mediated resistant gene *qnrB2* in 3 out of 200 poultry meat samples collected from markets in Ghana [37]. In Africa, evidence-based findings of plasmid-encoded genetic determinants associated with fluoroquinolone resistance in *Salmonella* strains are generally rare [28]. Few cases of *qnrB* and *qnrS* genes have been reported in South Africa [40] and Nigeria [8], respectively. Studies in Europe have reported on the increasing rate of *qnrB* resistant genes in several European countries (including Spain, Italy and Netherlands) and these were mostly from animal sources (predominantly chicken and turkey) [41]. The most commonly reported *qnrB* variant is *qnrB2* and it is usually harboured in *Salmonella* serovars Agona, Derby, Enteritidis, Hadar, London and Montevideo [41]. Although *qnrB* resistant genes are often restricted to animal populations, there is a potential chance of future global transmission to humans, as variant *qnrB19* has already been implicated in human *S*. Typhimurium infection in Netherlands and Italy [42, 43]. Studies in Scotland also revealed the presence of *qnrB* and *qnrS* from returning travellers from Egypt and Nigeria [39]. Another study conducted in United States reported on low prevalence of plasmid-mediated fluoroquinolone resistant genes, particularly *qnrS* [12, 44]. However, this study contrasts findings from some European (like Germany and Poland) and Asian countries, depicting regional differences in the prevalence of plasmid-mediated fluoroquinolone resistant genes [45].

To date, there are 9 variants of *qnrS* resistant gene (*qnrS1 to qnrS9*) identified, with *qnrS1* being the most predominant [46]. Previous studies showed that *qnrS1* alone was capable of conferring reduced susceptibility to ciprofloxacin even in the absence of *gyrA* mutation [47]. Again, findings from Hopkins et al.*,* in the United States demonstrated that quinolone resistant gene increased ciprofloxacin MIC to 0.38–0.78 μg/ml [13], giving credence to the fact that, *qnr* genes might confer full resistance in the near future with MIC ≥1 μg/ml if strong surveillance system is not established to control spread of these plasmid-encoded genes.

Other plasmid mediated fluoroquinolone resistant genes not screened in this study include *qnrC, qnrD, qnrVC,* aac(6′)-lb and plasmid-mediated enhanced efflux pump mechanisms by QepAB and OqxAB [48]. Studies show that the global prevalence of these genes are low [41]. Although *qnr* genes are usually associated with plasmid-encoded ESBL genes [10, 18], no ESBL gene was identified among the *qnrS* positive strains in this study.

Resistance of *Salmonella* to ciprofloxacin and other related fluoroquinolones has serious public health implications because this class of antimicrobials is commonly used to treat invasive forms of *Salmonella* infections. Mechanisms by these plasmid-mediated genetic determinants lead to low-level resistance that by itself does not exceed clinical breakpoint for susceptibility. However, it facilitates selection of higher level resistance and makes pathogens harbouring PMQR genes difficult to treat [48, 49]. The present study could not examine for the presence of other genetic determinants (such as *qnrC, qnrD, qnrVC*, *aac(6′)-Ib-cr,* and *qepAB* genes) responsible for plasmid-mediated fluoroquinolone resistance. Another limitation to this study was the inability to link the novel mutations (*gyrA* - Ile203Ser and Lys154Asn; and *gyrB* – Phe601Leu) to their involvement in reduced ciprofloxacin susceptibility/resistance within the QRDR. A suggested approach is to conduct a conjugational transfer experiment by introducing wild-type allele of the *gyrA* and *gyrB* genes into a broad host-range plasmid vector [15].

## Conclusion

This study reports for the first time plasmid-mediated fluoroquinolone resistant gene *qnrS* in *Salmonella* clinical isolates in Ghana. Nonsynonymous mutations (Asp87Gly, Glu133Gly and Ser83Tyr) which confer *Salmonella* reduced susceptibility to ciprofloxacin were also detected as reported in several studies [27, 29], with 3 other novel mutations likely to confer *Salmonella* resistance. We recommend surveillance systems to track the evolution of *Salmonella* plasmid-mediated resistant genes and to ensure proper use of antibiotics and control of severe infections.

## Data Availability

The datasets used and/or analysed during the current study are available from the corresponding author on reasonable request.

## Abbreviations

APH: Agogo Presbyterian Hospital
BA: Blood Agar
CA: Chocolate Agar
CHRPE: Committee for Human Research Publication and Ethics
CLSI: Clinical and Laboratory Standards Institute
EBI: European Bioinformatics Institute
ESBL: Extended Spectrum Beta-lactamase
ExPASY: Expert Protein Analysis System
KATH: Komfo Anokye Teaching Hospital
KCCR: Kumasi Centre for Collaborative Research in Tropical Medicine
KNUST: Kwame Nkrumah University of Science and Technology
MIC: Minimum Inhibitory Concentration
MRSA: Methicillin-resistant *Staphylococcus aureus*
PCR: Polymerase Chain Reaction
PMQR: Plasmid-mediated Quinolone Resistance
QRDR: Quinolone Resistance Determining Region
SIB: Swiss Institute of Bioinformatics
TSI: Triple Sugar Iron Agar
WHO: World Health Organisation
